# Can Incidental Gastric GISTs During Bariatric Surgeries Change the Primary Plan of Surgery? A Single Team Experience and a Systematic Review of Literature

**DOI:** 10.1007/s11695-024-07224-2

**Published:** 2024-04-30

**Authors:** Ahmed Abokhozima, Mohamed H. Zidan, Hashem Altabbaa, Ahmed Abo Elmagd, Mohammed Alokl, Fatmaelzahraa Fathy, Ahmed Amgad, Osama Al Shaqran, Mahmoud Hammad Eissa, Aliaa Selim

**Affiliations:** 1 Alexandria Main University Hospital, Alexandria University, AlexandriaAlexandria, 5372066 Egypt; 2 Alexandria University, Alexandria, 21526 Egypt; 3 Ekbal Hospital, Alexandria, Egypt; 4 Faculty of Medicine, Helwan University, Cairo, Egypt

**Keywords:** Bariatric surgery, GIST, Gastrointestinal stromal tumors, Classification of bariatric surgeries

## Abstract

As bariatric surgeries (BS) increase, more incidental findings are liable to be discovered. Incidental gastric gastrointestinal stromal tumors (GISTs) during BS can be found in around 0.7% of the cases. In this article, we have performed a systematic review of the literature and added our data to those of the review to review a conceptual treatment strategy to both improve patient outcomes and decrease the risk of overall cancer. With the rise of new bariatric techniques, we have proposed a new classification to BS to enhance our description of the treatment strategy.

**Graphical Abstract:**

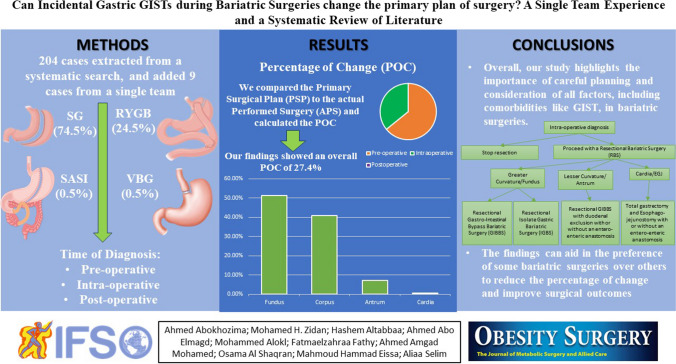

**Supplementary Information:**

The online version contains supplementary material available at 10.1007/s11695-024-07224-2.

## Introduction

Gastrointestinal stromal tumors (GISTs) are the most common mesenchymal tumors of the GI tract, mostly occurring in the stomach and small intestine [1–3]. They are generally solitary tumors, identified slightly more often in males than females of middle to advanced age [4]. GISTs have been identified during bariatric surgery procedures, and studies have indicated that the incidence of GISTs was reported to be more frequent in obese patients undergoing bariatric surgery (0.6–0.8%) as compared to the general population (0.001%) [5–7].

As the number of BS being carried out increases, more incidental findings such as GISTs, gastric polyps, and adenocarcinomas are identified. These findings may change the primary goal of the surgery, to improve the patient’s operative outcomes. In this article, we have reviewed all the cases of incidentally discovered gastric GISTs in a single bariatric center performed by a single bariatric team and added our findings to that of the literature. This is to evaluate the benefits of some bariatric procedures over others and determine the best course of action.

## Patients and Methods

### Patients Selected for Retrospective Analysis

We underwent a retrospective analysis of 2458 BS from January 2018 to January 2024 by a single bariatric team; 9 cases with incidental GISTs were found at the time of surgery and confirmed by pathology. During laparoscopy, the stomach was inspected thoroughly for tumor lesions. A CT scan was performed 5 months to 1 year after surgery, and an esophagogastric endoscopy was done after 1 year. Informed consent was obtained from all individual participants included in the study.

### Literature Search

**Fig. 1:**
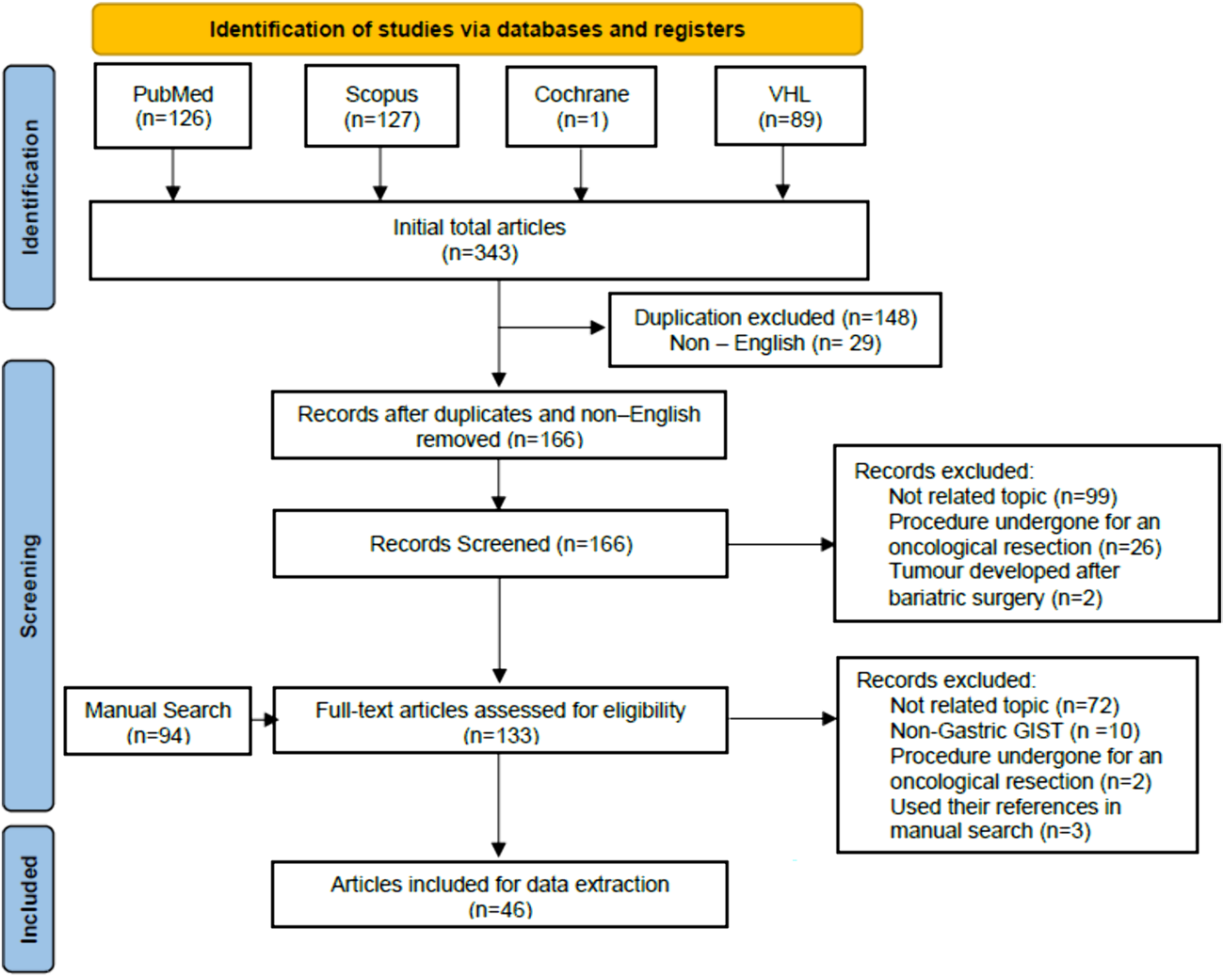
A PRISMA flow chart of our search through different databases

We conducted a systematic literature search between January 2005 and November 2023 using PubMed, ScienceDirect, Virtual Health Library, and Cochrane databases. The structure of the review was based on PRISMA guidelines [8]. The search terms used were (gastrointestinal stromal tumors or GIST) and (sleeve or bypass bariatric or gastrectomy). A total of 343 articles were identified from the search, and 94 articles were added through a manual search. We screened the articles for duplication and exclusion, and four authors performed data extraction, which four different authors validated. Duplicates and non-English articles were excluded during the first screening. The identified studies were also hand-searched to check for other relevant publications. We considered retrospective cohort studies, case series, case reports, reviews, and conference abstracts eligible for the study, given the rare occurrence of gastric GISTs in BS. Non-gastric GISTs were excluded from the data. After the screening and validation process, we included 46 articles for data analysis. A PRISMA flow chart was formulated (Fig. 1) to illustrate our search and data extraction methods.

Only cases that were presented to a bariatric clinic with morbid obesity and were incidentally discovered to have a gastric GIST were included. We have identified 204 cases of gastric GIST from 46 studies.

### Time of Diagnosis

Cases were classified into three groups according to the timing of diagnosis. Group A, patients who presented to a bariatric clinic that underwent a pre-operative diagnostic modality such as an endoscopy, that identified an incidental gastric GIST; Group B, patients with incidentally discovered GIST mass(es) intra-operatively and was confirmed by post-operative histopathology; Group C, GISTs diagnosed during the follow-up after BS by histopathology. Cases that presented with a gastric GIST and underwent a BS for a GIST were defined as non-incidental and were thus excluded.

### Classification of Bariatric Surgeries

To utilize our data, we have classified bariatric surgeries (BS) into either resectional bariatric surgeries (RBS) or non-resectional bariatric surgeries (NRBS). We defined RBS as any BS that involves resection of a significant part of the stomach, such as sleeve gastrectomy (SG), banded sleeve gastrectomy (BSG), single anastomosis sleeve ileal (SASI) bypass, single anastomosis duodenal-ileal bypass with sleeve (SADI-S), single anastomosis sleeve jejunal (SASJ) bypass, biliopancreatic diversion (BPD), and biliopancreatic diversion with duodenal switch (BPD-DS) bypass. NRBS was defined as any bariatric surgery that did not involve resection of the stomach, which included Roux-en-Y gastric bypass (RYGB), one-anastomosis gastric bypass (OAGB), vertical banded gastroplasty (VBG), adjustable gastric band (AGB), and jejunoileal bypass (JIB).

**Fig. 2:**
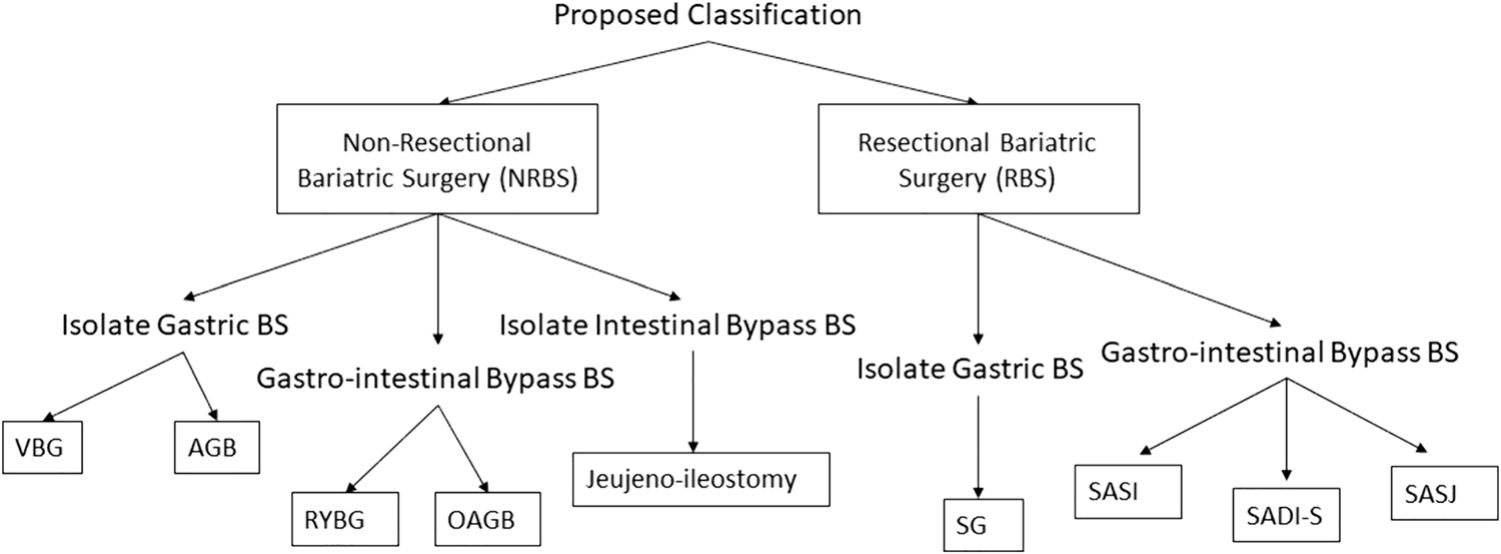
Our new proposed classification for bariatric surgeries (BS) into resectional bariatric surgery (RBS) or non-resectional bariatric surgery (NRBS). RBS is further divided into isolate gastric BS (IGBS) and gastrointestinal bypass BS (GIBBS); these include sleeve gastrectomy (SG), single anastomosis sleeve ileal bypass (SASI), single anastomosis sleeve jejunal bypass (SASJ), and single anastomosis duodenal-ileal bypass with sleeve (SADI-S). NRBS is divided into IGBS, GIBBS, and isolate intestinal bypass BS (IIBBS); these include vertical band gastroplasty (VBG), adjustable gastric band (AGB), Roux-en-Y gastric bypass (RYGB), one-anastomosis gastric bypass (OAGB), and jejunoileal bypass. Credits of this classification are to Mohamed H. Zidan

To further facilitate our discussion, we have further subdivided RBS and NRBS into isolated gastric bariatric surgeries (IGBS), gastrointestinal bypass bariatric surgeries (GIBBS), and isolated intestinal bypass bariatric surgeries (IIBBS). IGBS is defined as surgeries that only involve the stomach, and this can be either resectional, such as SG, or non-resectional, such as VBG and AGB. GIBBS is defined as BS that involves both the stomach and the small bowel by forming a gastro-enteric anastomosis to bypass a segment of the small bowel; these surgeries can be either resectional, such as SASI, SADI-S, SASJ, BPD, and BPD-DS, or non-resectional, such as RYGB or OAGB. IIBBS are defined as surgeries that only involve the small bowel by forming an entero-enteric anastomosis for diversion; these surgeries can only be non-resectional such as in JIB. Figure 2 illustrates our proposed classification. Credit was given to Mohamed H. Zidan for the formulation of this classification.

### Percentage of Change

We analyzed the primary surgical plan (PSP) of each case, compared it to the actual performed surgery (APS), and calculated the percentage of which the plan was changed or “the percentage of change” (POC). We defined PSP as the primary surgery aimed by the surgeon at the selected patient before routine pre-operative investigations (endoscopy) or the primary surgery pursued before discovering an incidental gastric GIST. The APS was defined as the performed surgery after pre-operative routine investigations or after discovering an incidental lesion. A change in the technique of surgery, either in the pre-operative or the intra-operative period, was defined as a change of plan from which we extracted the POC value.

We determined each PSP to the location of the GIST, compared it to the APS to the same location and determined whether the surgical plan had changed, further determining the POC of each location. The POC was also analyzed concerning the timing of the diagnosis in terms of the pre-operative and intra-operative setting and the type of bariatric surgery (RBS vs. NRBS).

### Analysis

Descriptive statistics were performed using SPSS, version 25.0. The data extracted from the articles selected and the records from our institute were added together and analyzed. Data are expressed as mean ± standard deviation. Patients were either diagnosed pre-operatively [9–15], intra-operatively [9, 14, 16–19, 4, 20–31], or post-operatively [6, 9, 11, 19, 26, 4, 32–50]. The PSP was analyzed and compared to the APS for the calculation of POC. We extracted the total number of bariatric patients from 37 articles that mentioned the number of patients. The incidence was calculated as the number of patients with GISTs diagnosed divided by the number of BS procedures performed.

We identified the country location (Supplementary File 1, Fig. S1) and year of publishing (Supplementary File 1, Fig. S2) of each article to observe the trend of research interest in our topic.

## Results

### Incidence

Out of 46 studies we analyzed, 204 patients were found to have gastric GIST undergoing bariatric surgery for obesity. This resulted in a total of 210 gastric GIST lesions, with 6 cases having 2 lesions. Our study identified 9 cases of solitary gastric GIST, bringing the total number of cases to 213 and the total number of GIST lesions to 219. Only 6 gastric GIST lesions (2.8%) were multiple lesions, while the majority of gastric GIST lesions were solitary (97.18%).

In total, 28,319 patients had undergone bariatric surgeries across 34 different institutes. However, only 19,664 cases were included in the studies, and we added our cases (2458) to calculate a rough denominator of 30,777 patients. It is important to note that 12 articles did not mention the total number of cases in their institutes. Based on our findings, the incidence of gastric GIST identified during bariatric surgery is less than 0.7% (Table 1).

**Table 1:** Descriptive parameters of all the cases included

	Review	Our study	Total cases
Number of bariatric patients in each institute	(*n* = 34 studies) 28,319	(*n*** = **1 study) 2458	(*n*** = **36 studies) 30,777
Prevalence			
Number of patients in all the studies	(*n* = 19,664)	(*n* = 9)	(*n* = 19,673)
Number of cases with gastric GIST	204 (1.04%)	9 (100%)	213 (1.09%)
Number of patients in all the institutes	(*n* = 28,319)	(*n* = 2458)	(*n* = 30,777)
Number of cases with gastric GIST	204 (0.7%)	9 (0.36%)	213 (0.7%)
Lesions per case	(*n* = 46 studies)	(*n* = 1 study)	(*n* = 47)
Cases with Gastric GIST	204	9	213
Number of Gastric GIST lesions	210 (*n* = 209)	9	219
Solitary Gastric GIST	198 (97.05%)	9 (100%)	207 (97.18%)
Multiple Gastric GIST	6 (2.94%)	0 (0%)	6 (2.81%)
Age	(*n* = 157)	(*n* = 9)	(*n* = 166)
Min–max	21 – 74	34 – 57	21 – 74
mean ± SD	50.81 ± 11.91	49.22 ± 7.412	50.72 ± 11.69
Sex	(*n* = 160)	(*n* = 9)	(*n* = 169)
Male	53 (33.1%)	3 (33.3%)	56 (33.1%)
Female	107 (66.9%)	6 (66.7%)	113 (66.9%)
BMI	(*n* = 151)	(*n* = 9)	(*n* = 160)
Min–max	23 – 72	39 – 56	23 – 72
Mean ± SD	44.3 ± 7.12	44.17 ± 5.512	44.3 ± 7.02
Size of GIST (mm)	(*n* = 175)	(*n* = 5)	(*n* = 180)
Min–max (mm)	1 – 91.13	7 – 20	1 – 91.13
Mean ± SD (mm)	10.94 ± 13.72	12.4 ± 5.13	10.98 ± 13.55
Location	(*n* = 170)	(*n* = 9)	(*n* = 180)
Fundus	89 (52.4%)	3 (33.3%)	92 (51.1%)
Corpus	68 (40%)	5 (55.6%)	73 (40.6%)
Antrum	12 (7.06%)	1 (11.1%)	13 (7.2%)
Cardia	1 (0.6%)	0 (0%)	1 (0.6%)
Primary surgical plan (PSP)	(*n* = 203)	(*n* = 9)	(*n* = 212)
LSG	150 (73.9%)	8 (88.9%)	158 (74.5%)
RYGB	52 (25.6%)	0 (0%)	52 (24.5%)
VBG	1 (0.5%)	0 (0%)	1 (0.5%)
LSASI	0 (0%)	1 (11.1%)	1 (0.5%)
Actual performed surgery (APS)	(*n* = 203)	(*n* = 9)	(*n* = 212)
LSG	150 (73.9%)	8 (88.9%)	158 (74.5%)
Laparoscopic Trans-gastric Resection with Concomitant LSG	1 (0.5%)	0 (0%)	1 (0.5%)
RYGB with the removal of all/part of the gastric pouch of the gastric pouch	49 (24.1%)	0 (0%)	49 (23.1%)
Endoscopic Submucosal resection and LSG	1 (0.5%)	0 (0%)	1 (0.5%)
Total gastrectomy	2 (1%)	0 (0%)	2 (1%)
LSASI	0 (0%)	1 (11.1%)	1 (0.5%)
Percentage of change (POC)	(*n* = 203) 58 (28.6%)	(*n* = 9) 0 (0%)	(*n* = 212) 58 (27.4%)
Follow-up	(*n* = 105)	(*n* = 9)	(*n* = 114)
Min–max	0.13 – 75	5 – 57	0.13 – 75
Mean ± SD	29.82 ± 18.06	18.66 ± 19.01	28.93 ± 18.3

### Demographics

We extracted age, sex, and BMI data from our search. Out of the 184 cases, we were able to determine age in 166 cases, sex in 169 cases, and BMI in 160 cases. The demographic data is presented in Table 1. The majority of the cases were female (66.9%) contradicting the existing literature [4]. Most age groups (21–74) had gastric GIST lesions, and the mean age of the patients was 50.72 ± 11.69. Although the BMI should be stated to be indicative of BS, the BMI range of our data fell between 23 and 72 kg/m^2^ with a mean of 44.3 ± 7.02 kg/m^2^. A single study reported a low value of 23 kg/m^2^ [4], and data was rechecked for validation of this value.

### Location and Size of the Lesions

**Fig. 3:**
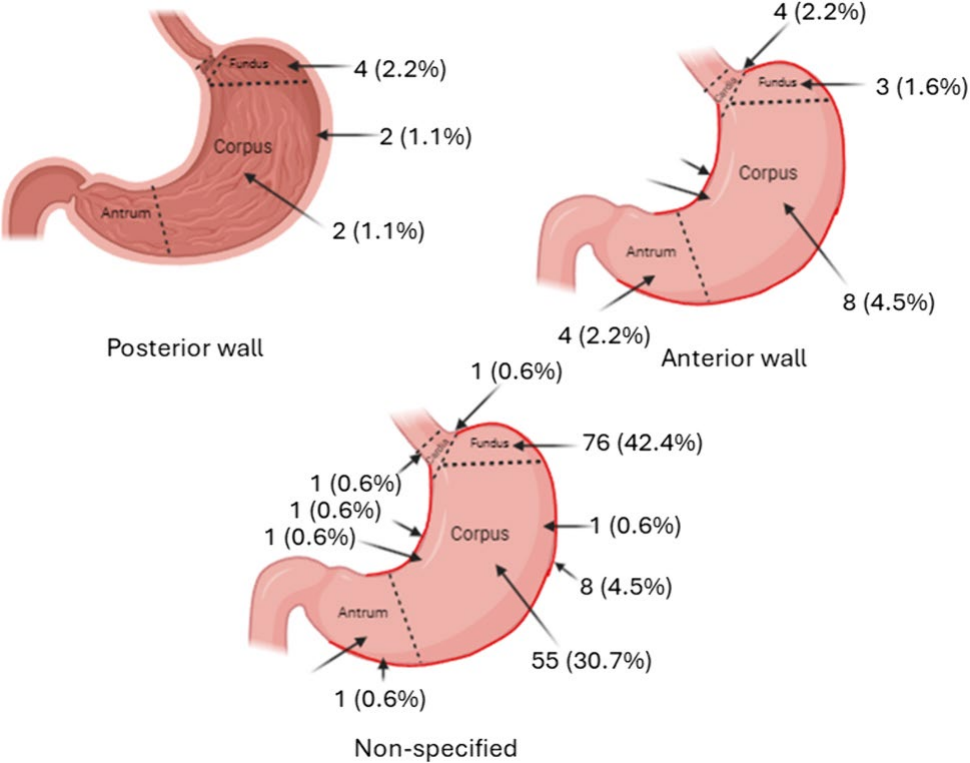
The figure shows the precise positions of gastric GISTs that were detected in both the review and retrospective analysis of our center. These positions are in relation to the anterior and posterior walls of the stomach. Any lesions that were not explicitly stated as either anterior or posterior were classified under the “non-specified” category

In our study, we determined the location and size of all the gastric GIST lesions in both our institute’s cases and the cases reviewed. Out of the 219 gastric GIST lesions, only 180 cases had their location in the stomach defined. We tried to detect the occurrence of gastric GIST in both the anterior and posterior surfaces; however, the data was insufficient as most of the GIST lesions were unspecified (Fig. 3). Lesions in the greater and lesser curvature were rounded to the closer segment to provide sufficient data for analysis. We rounded the exact locations to the “Fundus,” “Corpus,” “Antrum,” or “Cardia.” The incidence of each location is presented in Table 1.

We also recorded the size of the lesions in millimeters (mm) for the 180 gastric GIST cases. Our analysis found that the size of the lesions varied significantly, with the smallest being 1 mm and the largest measuring up to 91 mm. The mean size of the lesions was 11 ± 13.52 mm.

### Percentage of Change

We compared the primary surgical plan (PSP) to the actual performed surgery (APS) and calculated the percentage of change (POC). We analyzed the POC comparatively with the location of the GIST, the timing of diagnosis of GIST, the PSP, and the type of BS.

Our findings showed an overall POC of 27.4% (Table 1). The POC was calculated as the number of cases by which the PSP was changed either in the pre-operative setting (group A) or the intra-operative setting (group B). Group A had a POC of 60%, group B had a POC of 33.5%, and group C (post-operative diagnosis) showed no POC (0%) (Table 2).

**Table 2:** Percentage of change in each group and the detection methods used

Timing of Detection	Group A pre-operative	Group B intra-operative	Group C post-operative	Total
Detection method	(*n* = 10)	(*n* = 155)	(*n* = 48)	(*n* = 213)
Histopathology	1 (10%)	155 (100%)	48 (100%)	204 (95.8%)
EGD	9 (90%)	0 (0%)	0 (0%)	9 (4.3%)
Percent of change (POC)	(*n* = 213) 6 (2.8%)	(*n* = 213) 52 (24.4%)	(*n* = 213) 0 (0%)	(*n* = 213) 58 (27.2%)

We observed that the location of the GIST mass significantly impacted the percentage of change. The Fundus had a POC of 26.1%; the Corpus had a POC of 32.9%; the Antrum had a POC of 46.2%, and the Cardia with a POC of 100% (Table 3). We also calculated the POC for different surgical techniques, revealing that RBS had a low POC of 2.5%, and NRBS had a percentage of change of 100% (Table 4).

**Table 3:** Percentage of Change (POC) according to the location of the gastric GIST

Location of GIST	Primary surgical plan (PSP)	Actual performed surgery (APS)	Percentage of change (POC)		
			Number	POC of each location	POC valid percentage
Fundus (*n* = 92)	LSG (76.1%; *n* = 70)	LSG (74%; *n* = 68) RYGB with the removal of all/part of the gastric pouch of the gastric pouch (2.2%; *n* = 2)	24	26.1%	51.4%
	RYGB (23.9%; *n* = 22)	LSG (3.3%; *n* = 3) RYGB with the removal of all/part of the gastric pouch of the gastric pouch (19.6%; *n* = 18) Total gastrectomy (1.1%; *n* = 1)			
Corpus (*n* = 73)	LSG (65.7%; *n* = 48)	LSG (65.7%; *n* = 48)	24	32.9%	40.8%
	RYGB (32.9%; *n* = 24)	LSG (1.4%; *n* = 1) RYGB with the removal of all/part of the gastric pouch of the gastric pouch (31.5%; *n* = 23)			
	LSASI (1.4%; *n* = 1)	LSASI (1.4%; *n* = 1)			
Antrum (*n* = 13)	LSG (69.2%; *n* = 9)	LSG (46.15%; *n* = 6) RYGB with the removal of all/part of the gastric pouch of the gastric pouch (14.3%; *n* = 2) Endoscopic Submucosal resection and LSG (7.14%; *n* = 1)	6	46.2%	7.3%
	RYGB (28.6%; *n* = 4)	RYGB with the removal of all/part of the gastric pouch of the gastric pouch (28.6%; *n* = 4)			
Cardia (*n* = 1)	RYGB (100%; *n* = 1)	Total gastrectomy (100%; *n* = 1)	1	100%	0.6%
Total (*n* = 179)	LSG (70.9%; *n* = 127)	LSG (68.15%; *n* = 122) RYGB with the removal of all/part of the gastric pouch of the gastric pouch (2.2%; *n* = 4) Endoscopic Submucosal resection and LSG (0.5%; *n* = 1)	55	30.7%	100%
	RYGB (28.3%; *n* = 51)	LSG (2.2%; *n* = 4) RYGB with the removal of all/part of the gastric pouch of the gastric pouch (25%; *n* = 45) Total gastrectomy (1.1%; *n* = 2)			
	LSASI (0.55%; *n* = 1)	LSASI (0.55%; *n* = 1)

**Table 4:** Percentage of change (POC) of different primary surgical plans (PSP), resectional bariatric surgeries (RBS), and non-resectional bariatric surgeries (NRBS)

Primary surgical plan (PSP)	Percentage of change (POC)	Resectional bariatric surgery (RBS) vs. non-resectional bariatric surgery (NRBS)	POC
SG (*n* = 158)	4 (2.5%)	RBS	2.5%
SASI (*n* = 1)	0 (0%)		
RYGB (*n* = 52)	52 (100%)	NRBS	100%
VBG (*n* = 1)	1 (100%)		

## Discussion

Incidental findings during bariatric surgeries pose a challenge in our practice that is ignored in the literature. Few studies have mentioned these challenges, and they were not promoted enough in the literature. We, therefore, have tried to discuss this challenge through a precise study of incidental gastric GISTs during bariatric surgeries.

### Incidence and Demographics

GISTs are the most common mesenchymal tumors of the GI tract, which are found mainly in the stomach and small intestine [1–3]. These tumors are typically solitary and occur more frequently in males than females, especially in middle to advanced-age groups [4]. Our data showed that incidental gastric GISTs during bariatric surgeries are solitary in 97.18%; however, the most common sex was female; this might be because females are more likely to undergo BS than males [51].

Interestingly, studies have revealed that the incidence of GISTs is higher in obese patients undergoing bariatric surgery (0.6–0.8%) than in the general population (0.001–0.0015%) [5–7, 52]. Our data also agrees with this statement in that the incidence of incidental gastric GIST during BS is 0.7%. The greater incidence during BS than that of the general population ensures a strong correlation between GISTs with obesity [18].

### Pre-operative Work-up and Detection

The utilization of routine pre-operative endoscopy in BS is a long debatable subject in literature. Many studies have encouraged the usage of pre-operative endoscopy as a routine setting before surgery, as many findings are liable to change the plan of surgery or refute surgery from the start [15, 53, 54]. According to the 2009 SAGES (Society of American Gastrointestinal and Endoscopic Surgeons) guidelines [55], pre-operative endoscopy was recommended for all patients, especially those undergoing non-resectional GIIBBS. Moreover, the recent 2020 IFSO (International Federation for the Surgery of Obesity and Metabolic Disorders)[56] strengthened the recommendations for those patients with upper GI symptoms and those undergoing bypass and advised the usage of pre-operative endoscopy for all cases undergoing BS as there is a 25.3% chance of an unexpected finding that may alter management or contra-indicate surgery [56].

Our data shows that 143 patients from a total of 30,777 patients had a pre-operative endoscopy, out of which 10 patients were diagnosed with gastric GIST. However, 6 patients in the pre-operative group had changed their operative plan in the pre-operative setting, with a total POC of 2.8% (Table 2). Therefore, the efficiency of routine endoscopy to the POC is 4.2% (we calculated the number of cases with change of plan, *n* = 6, from the total cases of routine endoscopy, *n* = 143). In other words, there is a 4.2% chance that a routine pre-operative endoscopy would detect a gastric GIST and change the plan of surgery pre-operatively to help in a better setting. It is important to state that the number of cases that had undergone pre-operative endoscopy is low to determine, any strong recommendations to support or oppose the value of pre-operative endoscopy in detecting incidental gastric GIST. However, we believe that this percentage would increase significantly if more centers were encouraged to undergo routine pre-operative endoscopy.

Furthermore, the change of surgical plan was more determined and associated with far more innovative methods of management as endoscopic submucosal tunnel resection followed by LSG [13] and laparoscopic trans-gastric resection followed by LSG [12]. These findings from our review suggest that pre-operative endoscopy should be further encouraged, as it would improve the patient’s outcome by improving the planned surgical technique.

Our facility is familiar with the 2020 IFSO recommendations [56], and we do pre-operative endoscopy for symptomatic cases, patients undergoing re-do surgeries, and patients undergoing non-resectional GIBBS. However, we selectively do pre-operative endoscopy for other BS, and this is to limit the cost of care to the patients. Nevertheless, patients are provided the option of this procedure as well as post-operative histopathology.

A 2022 survey was conducted by Quake et al. on 121 respondents to determine whether surgeons followed the 2020 IFSO recommendations for pre-operative endoscopy [57]. The survey revealed that 53.7% (*n* = 65) of surgeons comply with this recommendation [57]. Furthermore, our data shows that only 143 patients (67.1%) had a routine pre-operative endoscopy. However, we believe that the actual number is much lower, especially in high-volume centers located in the Middle East and North Africa. This might be due to the low cost-effectiveness of this procedure. However, we do not have enough supporting data to substantiate our claim, and further studies should be conducted to both evaluate the cost-effectiveness of pre-operative endoscopy and to survey surgeons in different regions using the same standards provided by Quake et al. This will provide more accurate and efficient data as well as strengthen the recommendation for pre-operative endoscopy.

### Intra-operative Detection

A routine exhaustive intra-operative abdominal exploration during BS would theoretically increase the possibility of identifying incidental lesions. Some authors suggested this theory [58]; however, this technique would unnecessarily increase the operative time with increased morbidity risks [7]. However, exploration should be limited to the organs involved in the BS [16].

Inspection varies according to the surgical technique chosen. In RBS, such as SG, BSG, SASI, SADI-S, and SAS-J, an inspection of the anterior and the posterior gastric wall is done; furthermore, resectional GIBBS have an added value over resectional IGBS in that the small bowel is also inspected for bypass. Emile and Mahdy, for instance, recommend measuring the entire length of the small bowel before creating a common channel to decrease the risk of developing hypo-albuminemia [59]; this technique would further add value to resectional GIBBS in increasing the chances of detecting abnormal incidental gastric and small bowel pathologies.

Given the novelty of laparoscopic SASI, our conducted review dictates that there are no documented cases of incidental GIST during this procedure, making our case the first of its kind in the literature. A detailed account of the case presentation and management is available in video clip 1, which is included in this article for reference.

NRBS, on the other hand, have a lower exposure to the abdominal organs. Non-resectional IGBS have very limited exposure to the stomach and no exposure to the small bowel. Non-resectional GIBBS have very limited exposure to the stomach but have good exposure to the small bowel. Lastly, non-resectional IIBBS have no exposure to the stomach, but good exposure to the small bowel.

Our data states that NRBS have lower incidence rates of gastric GISTs; this might be due to the lack of exploration of the posterior gastric wall during dissection and the limited view of the stomach. On the other hand, RBS had a higher incidence of incidentally discovered gastric GISTs; this is due to the added value of post-operative histopathology and the extensive exploration of the gastric wall. Furthermore, NRBS correlated with higher POC, as all RYGB reported in the study had to undergo non-planned resection of part or all of the gastric remnant.

Although our data did not evaluate the importance of an exhaustive small bowel exploration for incidental small bowel GISTs, we agree with previous studies that this technique is important in GIBBS and IIBBS as a part of the procedure [16].

### Location, Size, and Management

The presence of GISTs and other incidental findings in the stomach does affect the surgical technique. Fernández et al. [7] provided a strong discussion on the possible management of incidental GISTs during bariatric surgeries. In our study, we have considered their recommendations and further classified bariatric surgeries into resectional (RBS) and non-resectional (NRBS) bariatric surgeries to gain a deeper insight into the recommended metabolic surgeries that can be undertaken in such instances.

To gain this recommendation, we depended on the POC calculated according to the type of surgery and the location of the GISTs in our study, and we then compared our findings to those of Fernández et al. [7]. It is important to note the bias of this comparison, as our study only analyzes gastric GIST lesions discovered during BS. In contrast, Fernández et al. have analyzed all GIST lesions detected during BS.

In the pre-operative setting, Fernández et al. recommended that if GIST is identified, the size of GIST is important to indicate the plan. Lesions > 2 cm had to be resected with or without a BS, while tumors < 2 cm were classified as either tumors that would alter the BS or lesions amenable to resection during BS. Lesions that would alter BS were recommended for either endoscopic management and follow-up or endoscopic management followed by a BS.

Fernández et al. also recommended that in the intra-operative setting, the surgeon can stop the surgery if resectability is questionable. However, if resection is to be undertaken, Fernández et al. suggested that the options should fall between LSG and RYGB according to the site and size of the lesion. Lesions in the greater curvature and the Fundus were recommended for LSG. However, lesions in the lesser curvature, depending on the size of the lesion where < 2 cm, were advised for wedge resection with LSG or RYGB with resection of the gastric remnant pouch. Lesions > 2 cm were advised for RYGB with resection of the gastric remnant pouch. Furthermore, lesions in the esophagogastric junction were recommended for gastrectomy and esophago-jejunal anastomosis. However, Fernández et al. recommended that during RYGB, if GIST was to be located in the gastric remnant, it is advised to either resect the remnant if the lesion is > 2 cm or perform a wedge resection if the lesion is < 2 cm.

**Fig. 4:**
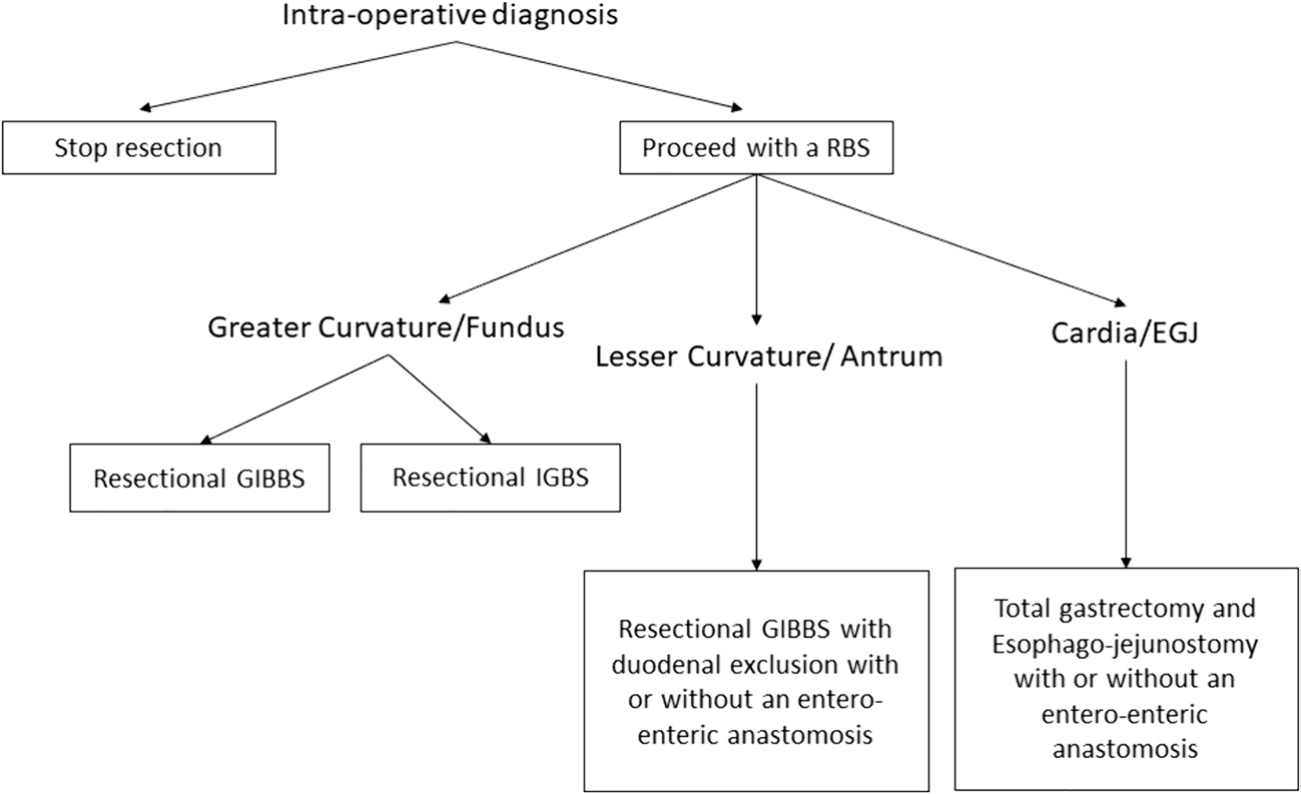
Algorithm of management of cases with intra-operative diagnosis of gastric GIST. Our algorithm is a modified version of that proposed by Fernández et al. [7], with the inclusion of our newly proposed classification (shown in Fig. 2). RBS: resectional bariatric surgery; GIBBS: gastrointestinal bypass bariatric surgery; IGBS: isolate gastric bariatric surgery

In the pre-operative setting, we agree with the recommendations of Fernández et al. [7]. However, in the intra-operative setting, we recommend that surgeons respect the site of the lesion before deciding on the next step. For lesions in the Fundus or greater curvature, we suggest performing either a resectional IGBS or a resectional GIBBS. Lesions in the lesser curvature or at the Antrum can proceed with a resectional GIBBS with a duodenal exclusion, with or without an entero-enteric anastomosis. For lesions in the Cardia or the esophagogastric junction, total gastrectomy and esophago-jejunostomy, with or without an entero-enteric anastomosis, are recommended. This is summarized in our algorithm in Fig. 4.

### Post-operative Work-up and Detection

The 2020 IFSO recommendations [56, 60] stated the importance of post-operative follow-up with endoscopy at 1 year and then every 2–3 years. This is highlighted in the theme of enabling early detection of Barrett’s esophagus, upper gastrointestinal (GI) malignancies, or other GI pathologies. This is important, especially in the background of OAGB, as there are concerns by many authors that OAGB might increase the risk of gastric and esophageal malignancies due to biliary reflux; however, there is no sufficient data to support this claim [61–66]. Furthermore, bypass surgeries have been reported to have a higher incidence of malignancies, especially in the excluded stomach (gastric remnant) [67], with many case reports highlighting the possibility of cancer development in the excluded stomach after both OAGB and RYGB [68–71].

On the other hand, BS is generally associated with a reduced risk of developing cancer [72–74]; however, this is correlational with the increased incidence of SG being performed. Wiggins et al. [72] noted that BS was associated with a significant reduction in overall cancer incidence compared to the overall population; however, in their study, 61.7% of the patients included had undergone LSG.

The reduction of overall cancer risk, with the majority of LSG being performed, raises the importance of RBS and can build a hypothesis for later studies for the evaluation of overall cancer risk following RBS and NRBS. We hypothesize that the risk of cancer reduction in the RBS group is higher than that of the NRBS group. However, further studies are needed to evaluate our statement before it can be taken into account. Furthermore, the importance of post-operative routine endoscopy should be highlighted more in literature to account for the development of metaplasia and dysplasia after BS.

Although post-operative endoscopy is encouraged to determine the incidence of GI pathologies, post-operative routine histopathology did not gain similar recommendations. Many studies, however, have stated the importance of post-operative histopathology, as many GI pathologies have been incidentally reported [43, 44, 75]. Furthermore, in our study, we have found that GIST was diagnosed post-operatively in 48 patients (22.5%) [6, 4, 9, 11, 19, 26, 32–50]; this can delineate the importance of routine post-operative histopathology. Therefore, we recommend that routine post-operative histopathology should be evaluated and encouraged after all RBS, as it would further reduce the overall risk of cancer.

### Limitations

Our systematic analysis and retrospective records only focused on the incidental finding of gastric GIST and did not evaluate the overall incidence of incidental findings or GISTs in other sites. Furthermore, our study did not compare the overall cancer risk of the proposed classification; however, we have only raised our hypothesis for further studies in these fields.

## Conclusion

With the increased rate of bariatric surgeries performed, an increased rate of incidental findings such as GISTs can be noted. With more and more findings being reported, it is important to change our perspective toward our current practices, as the use of RBS may increase the incidence of incidental findings that would further decrease the overall risk of cancer. Furthermore, more studies must discuss other valuable methods in managing incidental findings discovered during bariatric surgery.

It is important to conduct further studies to evaluate the risk of cancer development or reduction after both RBS and NRBS procedures, with short- and long-term follow-ups. This will allow us to gain a better understanding of the safety of these procedures and to incorporate any newer surgical techniques that are being introduced into our literature.

Additionally, more effort should be undertaken to highlight further the importance of pre and post-operative endoscopy as well as post-operative histopathology.

### Supplementary Information

Below is the link to the electronic supplementary material.

Supplementary file 1 (DOCX 94.8 KB)

Supplementary file 2 (MP4 44.4 MB)

## Data Availability

All data generated in this article is available in the article in Table 1, Table 2, Table 3, and Supplementary File 1.
