# Supplementary material for: Can Incidental Gastric GISTs During Bariatric Surgeries Change the Primary Plan of Surgery? A Single Team Experience and a Systematic Review of Literature
Source: Obes Surg. 2024 Apr 30;34(6):2186–97. doi: 10.1007/s11695-024-07224-2 (PMC11127811; doi:10.1007/s11695-024-07224-2)

**Supplementary tables and figures**

Table S1: Description of data included in our search and retrospective data of our institute.

|  | **Descriptive Statistics** | | | | | | |
| --- | --- | --- | --- | --- | --- | --- | --- |
|  | | N of study/patients | Sum | Min | Max | Mean | Std. Deviation |
| Number of patients in the study | | 46 | 19673 | 1 | 3052 | 409.98 | 663.540 |
| Number of patients in the institute (n) | | 36 | 30778 | 1 | 3052 | 854.94 | 813.420 |
| Number of cases with GIST | | 47 | 217 | 1 | 17 | 4.62 | 4.656 |
| Number of gastric GIST | | 47 | 213 | 1 | 17 | 4.53 | 4.648 |
| Number of masses | | 47 | 219 | 1 | 20 | 4.66 | 4.905 |
| Age | | 166 |  | 21 | 74 | 50.72 | 11.695 |
| BMI | | 160 |  | 23.70 | 72.00 | 44.3158 | 7.02429 |
| Follow-up period (m) | | 114 |  | .13 | 75.00 | 28.9350 | 18.30017 |
| Size of the GIST (mm) | | 180 |  | 1.00 | 91.13 | 10.9851 | 13.55152 |

Table S2: Cases extracted from our search and retrospective data of our institute.

|  | **Case Processing Summary** | | | | | | |
| --- | --- | --- | --- | --- | --- | --- | --- |
|  | | N of Gastric GIST | | Not gastric GIST | | Total | |
|  | | N | Percent | N | Percent | N | Percent |
| Number of patients in the study | | 213 | 1.1% | 19460 | 98.9% | 19673 | 100.0% |
| Number of patients in the institute (n) | | 213 | 0.7% | 30565 | 99.3% | 30778 | 100.0% |

Table S3: Sex identified from our search and retrospective data of our institute.

| **Sex** | | | | | |
| --- | --- | --- | --- | --- | --- |
|  | | Frequency | Percent | Valid Percent | Cumulative Percent |
| Valid | Male | 56 | 26.3 | 33.1 | 33.1 |
|  | Female | 113 | 53.1 | 66.9 | 100.0 |
|  | Total | 169 | 79.3 | 100.0 |  |
|  | N/A | 44 | 20.7 |  |  |
| Total | | 213 | 100.0 |  |  |

Table S4: Timing of diagnosis of the cases from our search and retrospective data of our institute.

| **Time of diagnosis** | | | | | |
| --- | --- | --- | --- | --- | --- |
|  | | Frequency | Percent | Valid Percent | Cumulative Percent |
| Valid | Pre-operative | 10 | 4.7 | 4.7 | 4.7 |
|  | Intra-operative | 155 | 72.8 | 72.8 | 77.5 |
|  | Post-operative | 48 | 22.5 | 22.5 | 100.0 |
|  | Total | 213 | 100.0 | 100.0 |  |

Table S5: Primary surgical plan of the cases from our search and retrospective data of our institute.

| **Primary surgical plan** | | | | | |
| --- | --- | --- | --- | --- | --- |
|  | | Frequency | Percent | Valid Percent | Cumulative Percent |
| Valid | LSG | 158 | 74.2 | 74.5 | 74.5 |
|  | RYGB | 52 | 24.4 | 24.5 | 99.1 |
|  | VBG | 1 | .5 | .5 | 99.5 |
|  | LSASI | 1 | .5 | .5 | 100.0 |
|  | Total | 212 | 99.5 | 100.0 |  |
|  | N/A | 1 | .5 |  |  |
| Total | | 213 | 100.0 |  |  |

Table S6: Intraoperative management plan conducted in the cases from our search and retrospective data of our institute.

| **Intra-operative management** | | | | | |
| --- | --- | --- | --- | --- | --- |
|  | | Frequency | Percent | Valid Percent | Cumulative Percent |
| Valid | LSG | 158 | 74.2 | 74.5 | 74.5 |
|  | Laparoscopic Trans-gastric Resection with Concomitant LSG | 1 | .5 | .5 | 75.0 |
|  | RYGB with the removal of all/part of the gastric pouch of the gastric pouch | 49 | 23.0 | 23.1 | 98.1 |
|  | Endoscopic Submucosal resection and LSG | 1 | .5 | .5 | 98.6 |
|  | total gastrectomy | 2 | .9 | .9 | 99.5 |
|  | LSASI | 1 | .5 | .5 | 100.0 |
|  | Total | 212 | 99.5 | 100.0 |  |
|  | N/A | 1 | .5 |  |  |
| Total | | 213 | 100.0 |  |  |

Table S7: H.pylori Tests conducted in cases from our search and retrospective data of our institute.

| **H-Pylori test** | | | | | |
| --- | --- | --- | --- | --- | --- |
|  | | Frequency | Percent | Valid Percent | Cumulative Percent |
| Valid | Positive | 21 | 9.9 | 28.4 | 28.4 |
|  | Negative | 53 | 24.9 | 71.6 | 100.0 |
|  | Total | 74 | 34.7 | 100.0 |  |
|  | N/A | 139 | 65.3 |  |  |
| Total | | 213 | 100.0 |  |  |

Table S8: Location of the gastric GISTs identified from cases in our search and retrospective data of our institute.

| **Location of the GIST** | | | | | |
| --- | --- | --- | --- | --- | --- |
|  | | Frequency | Percent | Valid Percent | Cumulative Percent |
| Valid | Corpus | 73 | 33.3 | 40.8 | 40.8 |
|  | Fundus | 92 | 42.0 | 51.4 | 92.2 |
|  | Antrum | 13 | 5.9 | 7.3 | 99.4 |
|  | cardia | 1 | .5 | .6 | 100.0 |
|  | Total | 179 | 81.7 | 100.0 |  |
|  | N/A | 40 | 18.3 |  |  |
| Total | | 219 | 100.0 |  |  |

Figure S1: Simple Bar chart denoting the countries from which the articles were published mentioning gastric GISTs.


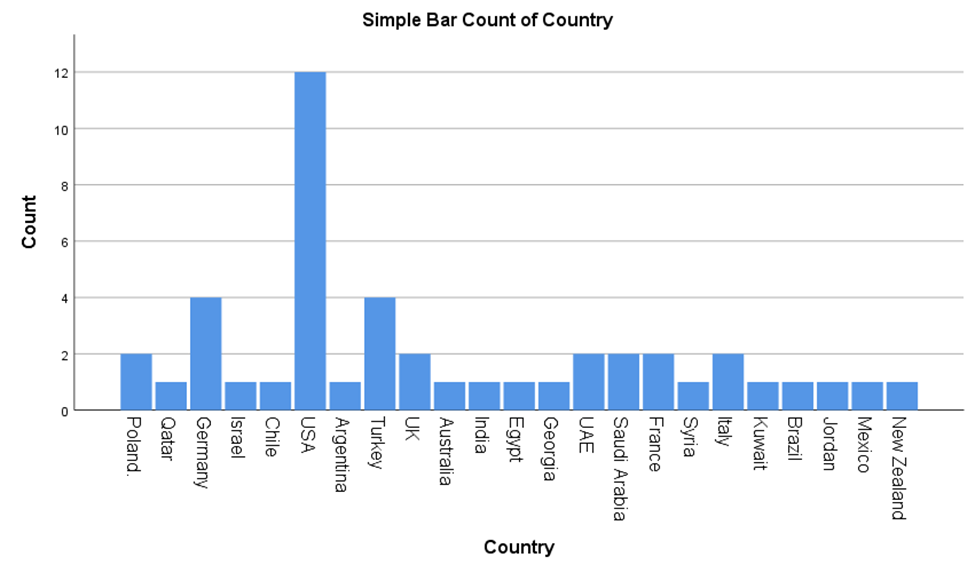


Figure S2: Years by which articles mentioning incidental gastric GISTs were published.


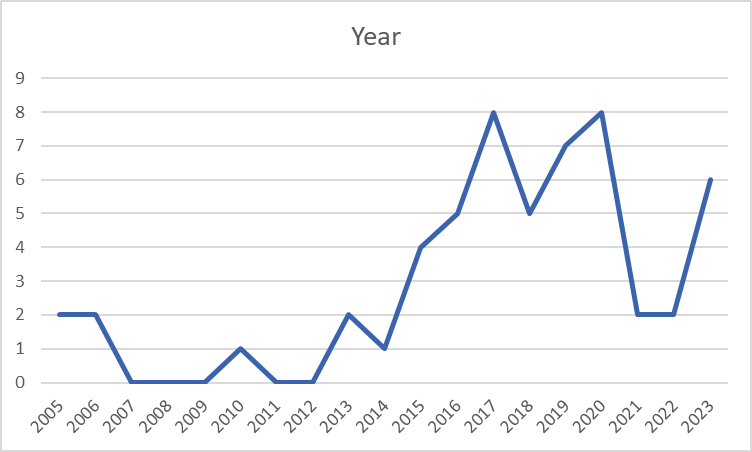

Supplement: Supplementary file 1 — (DOCX 94.8 KB) [file 11695_2024_7224_MOESM1_ESM.docx]
